# Predictive Power of Self-Rated Health for Subsequent Mortality Risk During Old Age: Analysis of Data From a Nationally Representative Survey of Elderly Adults in Taiwan

**DOI:** 10.2188/jea.JE20100131

**Published:** 2011-07-05

**Authors:** Christy Pu, Gao-Jun Tang, Nicole Huang, Yiing-Jenq Chou

**Affiliations:** 1 Institute of Hospital and Health Care Administration, School of Medicine, National Yang-Ming University, Taipei, Taiwan; 2 National Yang-Ming University Hospital, Yi-Lan, Taiwan; 3 Department of Public Health, School of Medicine, National Yang-Ming University, Taipei, Taiwan

**Keywords:** socioeconomic status, self-rated health, mortality, elderly

## Abstract

**Background:**

Previous research has investigated differences in the predictive power of self-rated health (SRH) for mortality based on socioeconomic status (SES). However, these studies mainly assessed adults in the general population and did not focus specifically on elderly adults. In addition, this predictive power has never been evaluated using subjective SES, which is an important measure of SES in elderly adults.

**Methods:**

This study used data from the Survey of the Health and Living Status of the Middle Aged and the Elderly in Taiwan (SHLS) conducted by the Bureau of Health Promotion, Taiwan. The SHLS is a 15-year longitudinal survey based on a nationally representative sample. It was initiated in 1989 with 4049 respondents aged 60 years or older. Both education and subjective financial satisfaction were used as SES measures in the present study. A Cox regression model was used to estimate the interaction between SRH and SES for 3829 individuals without missing data.

**Results:**

As compared with those who reported their health as good, those who reported their health as poor and their education as high had a higher hazard ratio (hazard ratio = 1.97, 95% confidence interval = 1.35–2.88) for 6–15-year mortality, after adjusting for depressive symptoms, activities of daily living, and instrumental activities of daily living. This HR was significantly higher than those for adults with middle (1.16, 0.93–1.44) and low (1.27, 1.05–1.54) education, based on the χ^2^ test (*P* < 0.05 for both comparisons). A similar pattern was observed when financial satisfaction was used as the SES measure. However, the pattern was attenuated when using 5-year mortality from baseline.

**Conclusions:**

The use of SRH as a single health measure in elderly adults may yield inconsistent results across different SES groups, especially when used as a predictor of a longer-term mortality. This is true regardless of whether objective or subjective measures of SES are used, where both are important measures of SES in elderly adults.

## INTRODUCTION

Self-rated health (SRH) is often used as a health measure in research to investigate the socioeconomic gradient. Its popularity lies in its predictive power in terms of future mortality.^1^^–^^3^ The power of SRH to predict subsequent mortality is strong in both Western and Asian populations.^4^^–^^7^ Previous studies have evaluated the predictive power of SRH on subsequent mortality based on markers of socioeconomic status (SES), including education, income, and occupation.^1^^–^^3^^,^^8^^–^^10^ However, very few of these studies focused on elderly adults. Because elderly populations have special characteristics with respect to SES,^11^ results from studies of younger cohorts should not be generalized to older age groups.

For SRH to be a valid measure in SES gradient research, it is often assumed that discrepancies between measured and true health status are equal among different socioeconomic groups.^2^ This was shown to be untrue for non-elderly adults. In addition, the predictive power of SRH for mortality is more accurate among people with a higher SES. For example, in a sample of Dutch adults aged 25 to 74 years, Huisman et al^2^ found that the predictive power was greater for men with tertiary education as compared with men with the lowest education. Similarly, in the United States, Dowd and Zajacova^1^ found that poorer SRH was more strongly associated with mortality among adults aged 25 years or older with a higher education level and a higher income than among those with lower SES. However, Singh-Manoux et al,^3^ in a sample with an average age of 44.2 years, found that the predictive ability of SRH for mortality was weaker for the higher SES group among middle-aged French adults.

The elderly population has several unique characteristics. Thus, analysis of the predictive power of SRH for mortality must be carried out using a sample that consists solely of elderly adults. First, the factors associated with a person’s health rating may differ in old age. For example, when assessing their health, people over 60 years of age tend to compare their health with that of others their own age; younger respondents may not do this.^12^ In addition, retirement may affect SRH.^13^ Whether these factors interact with SES during old age is unknown. Second, there has been continuing debate on whether SES disparities in health persist during old age. One of the most discussed factors is selective survival, whereby those who are most disadvantaged die earlier, which might dilute the effect of SES on health for elderly adults.^14^ Third, public health services provided during old age may reduce the role played by SES.^15^ Nevertheless, some authors argue that SES disparities may actually widen during old age, thereby supporting a framework of cumulative disadvantage.^16^ The debate is yet to be resolved.^14^^,^^17^^,^^18^

What measures are appropriate for SES during old age is another issue of discussion. Traditional SES measures may not be relevant for elderly adults, since income and occupation may not be good measures for this population, as they are retired and because of other predetermined conditions. In addition, the benefits of education may not be the same as those obtained from other life experiences.^11^ For these reasons, some researchers have proposed the use of subjective SES measures such as financial hardship and financial strain.^11^^,^^19^ Thus, the predictive power of SRH for mortality in terms of SES needs to be evaluated among elderly adults using these alternative SES measures, which was not the method used in previous studies.

Given the possibly inconsistent effect of SES on health during old age, it may be that there are disparities in terms of the predictive effect of SRH for mortality relative to SES during old age. We used a nationally representative sample of elderly adults with a mean age of 68.2 years to investigate whether the predictive power of SRH for mortality varied with SES during old age.

## METHODS

### Data

We used data from the Survey of the Health and Living Status of the Middle Aged and the Elderly in Taiwan (SHLS) conducted by the Bureau of Health Promotion (BHP), Department of Health, Taiwan, which is a 15-year longitudinal survey based on a nationally representative sample. The survey began in 1989 with 4049 respondents aged 60 years or older. The sampling framework was designed to ensure that the sample was representative of the elderly population in Taiwan. The sample includes both elders in the community and those living in institutions. It is particularly important to include those living in institutions (such as nursing homes) when studying elderly adults because it is more common for elderly adults to be in such institutions as compared with younger adults. A detailed description of the sampling method has been published elsewhere.^20^ The same cohort was followed up in 1993, 1996, 1999, and 2003. Data from all these years were included in the present analysis.

The SHLS is a publicly available dataset. It consists of detailed information on the demographics, socioeconomics, and lifestyle, as well as health status, of elderly adults in Taiwan. The survey was linked to the Death Registration Records by the Department of Health before releasing the data. Individual IDs are scrambled before data are publicly released to ensure that private information is protected. The standard procedure for obtaining access to the dataset consists of submitting a research protocol together with an application form to the BHP. The dataset is then released to the applicant after the application is reviewed by appropriate BHP staff. The feasibility of the study is the main consideration during the review process. Because all individuals in the dataset are anonymous and it is not possible to identify specific individuals from the dataset, the ethical review board of the BHP does not require the applicant to submit confirmation of ethical approval for use of the dataset.

Of the 4049 individuals at baseline, 143 had missing values for SRH. Of these 143 adults, 14 were alive at the time of the final wave (2003). Another 77 individuals had at least 1 missing value for the variables used, as described below. Ultimately, data from 3829 individuals were included in the analysis.

### Measures

All measures other than mortality were drawn from the baseline wave (1989). Survival status was obtained at all subsequent waves, up to 2003. The database was linked to national death records; thus, the year of death was available even if death occurred outside the years of the follow-up surveys. Time to death was therefore measured in years.

### Education and financial satisfaction

For education, the education status of respondents was initially classified as illiterate, literate with no formal education, primary school, junior high school, senior high school, and college or higher (Table 1). However, due to the characteristics of the sample (most subjects had low education), the education variable was reclassified as low (illiterate), medium (literate without formal education, and primary school), and high (junior high school or higher) to ensure a more even distribution across groups. At baseline, the respondents were asked “Are you satisfied with your financial position?”, to which they responded using a 5-point Likert-type scale ranging from very satisfied to very unsatisfied (Table 1). The purpose of this question was to measure the subjective financial position of the respondent. To ensure that there was a sufficient number of subjects within each category, we combined adjacent categories to form 3 groups (satisfied, average, dissatisfied).

**Table 1.** Distributions of study variables and proportions of deaths during 15-year follow-up according to categories of study variables

	Distribution at baseline, %	Proportion of deaths^a^
*n*	3829	1961
Age, years		
60–70	69.78	40.8
70–80	25.93	73.0
>80	4.28	88.4
Sex (male)	57.3	54.6
Education		
Illiterate	40.5	57.7
Literate with no formal education	8.8	52.8
Primary school	31.3	48.5
Junior high school	8.4	40.7
Senior high school	5.9	40.8
College or above	5.1	42.8
Financial satisfaction		
Very satisfied	9.9	44.7
Satisfied	33.5	48.9
Average	39	52.6
Dissatisfied	15.4	55
Very dissatisfied	2.3	65.1
Self-rated health		
Very good	17.5	38.4
Good	22	45.9
Fair	38.2	51.8
Poor	18.4	62.6
Very poor	3.9	78.6
Marital status		
Single/widowed	37	59.2
Married/common law marriage	63	46.5
Ethnicity		
Fuchien	60.6	53.6
Hakka	14.8	53.5
Mainlander	22.9	42.4
Other	1.6	64.5
CESD-17 score^b^		
≦15	82.1	49.3
>15	17.9	59.6
ADL score^c^		
≦2	51.7	41.3
>2	48.3	61.9
IADL score^c^		
≦2	82.7	46.4
>2	17.3	73.9
Number of people in household		
<2	9.9	58
2–5	49	47.4
>5	41.2	54.1

^a^Percentages represent the proportions of participants who died, by category.

^b^CESD: Center for Epidemiological Studies-Depression Scale.

^c^ADL and IADL stand for activities of daily living and instrumental activities of daily living, respectively.

### Self-rated health

SRH was used to measure the participants’ subjective health status. At the baseline survey, each respondent was asked the question “How would you rate your general health?”, for which the responses were very good, good, fair, poor, or very poor.

### Statistical analysis

The association between SRH and mortality was analyzed using Cox regression and hazard ratios (HRs). The Breslow approximation^21^ was used in cases of tied event times. To test whether the association varied by socioeconomic group (education and subjective financial position), a series of interactions were tested. To ensure that our results were comparable with those obtained from nonelderly adults, we followed the statistical methods employed by Dowd and Zajacova.^1^ The interactions were defined as the product of the 2 variables of interest. SRH was dichotomized into respondents who reported poor health and those who did not (poor/very poor health versus excellent/very good/fair health). Different groups were created based on level of education/financial satisfaction multiplied by their health status. The reference category was thus those reporting excellent/very good/fair health in the same educational/financial satisfaction category. Higher HRs indicate greater predictive power of SRH for mortality.^2^ χ^2^ tests for the simple effect of the interaction terms were performed to assess whether the HR for a particular SRH/SES group was significantly higher than the HRs of other groups.^22^ For sensitivity analysis, we re-estimated the models using SRH as a continuous variable^2^ and the original 5 ordered categories. The results were very similar to the dichotomized analysis (data not shown).

A total of 4 models were created. Model 1 controlled only for baseline characteristics, including baseline age, sex, household size (number of people living together), and ethnicity (Fuchien, Hakka, Mainlander, or other). Model 2 additionally controlled for depressive symptoms, which were measured using the Center for Epidemiological Studies-Depression Scale (CES-D 17-item), which consists of 17 items such as “I am bothered by things that usually don’t bother me”, “I do not feel like eating; my appetite was poor”, and “I feel that I cannot shake the blues even with help from my family or friends”. Each response consisted of a 4-point ordinal scale and thus has a maximum value of 51. The purpose of testing the predictive power of SRH on mortality was to determine whether SRH reflects the “true” health status of the participant. Thus, we investigated whether including activities of daily living (ADL) and instrumental activities of daily living (IADL) attenuated the predictive power (model 3). These 2 measures were also included to determine the actual physical health of the participants. ADL impairments was assessed using 9 questions, including inability to climb stairs, walk 200 meters, do housework, take a bus by oneself, lift a weight, bend down, lift arms over one’s head, use hands to take or twist lids or other items, and stand for 2 hours. The IADL included 4 items, including being unable to bathe, make phone calls, handle money, and buy groceries.^23^ For each of the above questions, the respondent could choose from a 4-point ordinal scale ranging from no difficulties (0 points) to total inability (3 points). These points are additive, so a higher total score indicates more severe disability. Model 4 further controlled for depressive symptoms, in addition to the variables in model 3.

It is reasonable to assume that the power of SRH to predict mortality by education level varies with the subject’s closeness to death. To test whether this was the case in our sample, we first assessed whether our Cox models satisfied the proportional hazards (PH) assumption, using the entire study period, ie, 1989–2003 (data not shown). The results of these tests showed that none of the models fulfilled the PH assumption, which indicates that the effects of the explanatory variables for mortality are not constant across different durations of follow-up. It is thus inappropriate to use the full follow-up period in a single Cox model. We therefore separated the analysis into 2 nonoverlapping intervals, 1989–1993 (hereafter 5-year mortality) and 1994–2003 (hereafter 6–15-year mortality). All models in each respective interval satisfied the PH assumption. All analyses were carried out using STATA/MP-10.1.^24^

## RESULTS

Table 1 shows the distribution of study variables and the proportions of participants who died during 15-year follow-up, according to categories of the study variables. Overall, 22.3% of the participants reported poor/very poor health at baseline. Approximately 51.2% of the sample died during the study period. The sample of elderly used in the present study had a low education level: 40.5% of the sample were illiterate and only 19.4% of the subjects were educated to junior high school level or higher. Of those who were illiterate, 57.7% died during follow-up. This percentage was higher than that of groups with a higher education level. Regarding SRH, the mortality rate was higher among those that reported poorer health. For example, among those who reported very poor health, 78.6% died during follow-up, which is more than double the 38.4% who reported very good health. Most subjects had a CES-D score of 15 or lower and a lower mortality rate during follow-up (49.3%) as compared with those with a CES-D score higher than 15 (59.6%). Those with greater ADL and IADL impairments (indicated by higher scores) had a higher mortality rate during follow-up. Mortality was also higher (58%) among those living with fewer than 2 people.

Tables 2 and 3 show the relationship between SRH and mortality with respect to education level and financial satisfaction, respectively. Among those with a high education level and poor self-reported health (*n* = 78), 74.4% died during follow-up; the corresponding figures for those with middle and low levels of education (and poor self-reported health) were 58.7% and 68.6%, respectively. This U-shaped relationship was less obvious for financial satisfaction; however, those with poor self-rated health and the highest financial satisfaction still had the highest mortality rate. In Tables 2 and 3, regression analyses were stratified by 2 nonoverlapping intervals, as described in the Method section. In the 6–15-year model, education was a significant predictor of mortality (the main effect), and participants with a low education level were more likely to die (HR, 1.22–1.42 in the 4 models). In all 4 models, the interactive effect showed that poor SRH was a stronger predictor of subsequent mortality among elderly adults with higher education levels. In model 1, among participants with a high level of education, the HR for those reporting poor health as compared with those reporting better health was 2.39 (95% CI = 1.64, 3.47), which was significantly higher than the HRs of 1.31 (1.06, 1.62) and 1.51 (1.25, 1.82) for those with middle and low education levels, respectively. Controlling for depressive symptoms (model 2) did not change the results. After controlling for ADL and IADL (model 3), there was a slight decrease in the effect, but the results were similar. A similar pattern was also found in model 4. Adjustment for either or both the physical and mental health domains thus did not alter the principal findings of the education models. The results, however, were different when the analysis was limited to a 5-year follow-up. In all 4 models, the HR for death was still higher among those with a high education level, but not significantly higher than the HRs for middle and low education groups.

**Table 2.** Hazard ratios for the main effect of education and interactions between education and self-rated health in relation to mortality (*n* = 3829)

Education	Self-rated health	*n*	Proportion of deaths during 15-year follow-up	5-year mortality								6–15-year mortality							
	
				Hazard ratio (95% confidence interval)								Hazard ratio (95% confidence interval)							
	
				Model 1		Model 2		Model 3		Model 4		Model 1		Model 2		Model 3		Model 4	
High	All	743	41.3	1.00	(referent)	1.00	(referent)	1.00	(referent)	1.00	(referent)	1.00	(referent)	1.00	(referent)	1.00	(referent)	1.00	(referent)
Middle	All	1537	49.5	1.01	(0.73, 1.39)	1.01	(0.73, 1.39)	0.99	(0.72, 1.36)	0.99	(0.72, 1.36)	1.23	(1.03, 1.47)	1.23	(1.03, 1.47)	1.22	(1.02, 1.46)	1.22	(1.02, 1.46)
Low	All	1549	57.7	1.32	(0.95, 1.85)	1.32	(0.95, 1.85)	1.24	(0.89, 1.74)	1.23	(0.88, 1.73)	1.42	(1.17, 1.73)	1.42	(1.17, 1.73)	1.35	(1.11, 1.64)	1.36	(1.12, 1.65)

High	Good	665	37.4	1.00	(referent)	1.00	(referent)	1.00	(referent)	1.00	(referent)	1.00	(referent)	1.00	(referent)	1.00	(referent)	1.00	(referent)
	Poor	78	74.4	3.33	(2.05, 5.42)	3.33	(2.05, 5.42)	2.21	(1.35, 3.63)	2.16	(1.32, 3.53)	2.39^a,b^	(1.64, 3.47)	2.39^a,b^	(1.64, 3.47)	1.89^a,b^	(1.30, 2.75)	1.97^a,b^	(1.35, 2.88)
Middle	Good	1215	47.0	1.00	(referent)	1.00	(referent)	1.00	(referent)	1.00	(referent)	1.00	(referent)	1.00	(referent)	1.00	(referent)	1.00	(referent)
	Poor	322	58.7	2.24	(1.66, 3.02)	2.24	(1.66, 3.02)	1.69	(1.24, 2.30)	1.67	(1.23, 2.25)	1.31	(1.06, 1.62)	1.31	(1.06, 1.62)	1.12	(0.91, 1.39)	1.16	(0.93, 1.44)
Low	Good	1094	53.2	1.00	(referent)	1.00	(referent)	1.00	(referent)	1.00	(referent)	1.00	(referent)	1.00	(referent)	1.00	(referent)	1.00	(referent)
	Poor	455	68.6	2.33	(1.81, 2.99)	2.33	(1.81, 2.99)	1.67	(1.29, 2.17)	1.64	(1.28, 2.10)	1.51	(1.25, 1.82)	1.51	(1.25, 1.82)	1.23	(1.02, 1.48)	1.27	(1.05, 1.54)

Model 1 was adjusted for baseline age, sex, ethnicity, marital status, and number of people living in household.

Model 2: model 1 additionally controlled for depressive symptoms.

Model 3: model 1 additionally controlled for ADL and IADL.

Model 4: model 2 additionally controlled for ADL and IADL.

*n = 3275 for the 6–15-year model.

^a^Significant difference between high and middle education groups based on the χ^2^ test.

^b^Significant difference between high and low education groups based on the χ^2^ test.

**Table 3.** Hazard ratios for the main effect of education and interactions between financial satisfaction and self-rated health in relation to mortality (*n* = 3829)

Financial satisfaction	Self-rated health	*n*	Proportion of deaths during 15-year follow-up	5-year mortality								6–15-year mortality							
	
				Hazard ratio (95% confidence interval)								Hazard ratio (95% confidence interval)							
	
				Model 1		Model 2		Model 3		Model 4		Model 1		Model 2		Model 3		Model 4	
Satisfied	All	1659	47.9	1.00	(referent)	1.00	(referent)	1.00	(referent)	1.00	(referent)	1.00	(referent)	1.00	(referent)	1.00	(referent)	1.00	(referent)
Average	All	1493	52.6	1.17	(0.93, 1.47)	1.16	(0.92, 1.47)	1.14	(0.91, 1.44)	1.14	(0.90, 1.43)	1.08	(0.94, 1.23)	1.08	(0.95, 1.23)	1.06	(0.93, 1.20)	1.06	(0.93, 1.21)
Dissatisfied	All	677	56.3	1.23	(0.88, 1.73)	1.22	(0.87, 1.72)	1.18	(0.84, 1.65)	1.17	(0.84, 1.62)	1.15	(0.95, 1.40)	1.16	(0.96, 1.41)	1.12	(0.93, 1.34)	1.13	(0.93, 1.38)

Satisfied	Good	1432	44.9	1.00	(referent)	1.00	(referent)	1.00	(referent)	1.00	(referent)	1.00	(referent)	1.00	(referent)	1.00	(referent)	1.00	(referent)
	Poor	227	67.0	2.71	(2.00, 3.67)	2.71	(2.00, 3.67)	1.90	(1.39, 2.60)	1.89	(1.39, 2.58)	1.71	(1.35, 2.15)	1.71	(1.36, 2.16)	1.44^a^	(1.14, 1.82)	1.46^a^	(1.15, 1.85)
Average	Good	1158	49.1	1.00	(referent)	1.00	(referent)	1.00	(referent)	1.00	(referent)	1.00	(referent)	1.00	(referent)	1.00	(referent)	1.00	(referent)
	Poor	335	64.8	2.40	(1.83, 3.17)	2.41	(1.83, 3.17)	1.84	(1.39, 2.44)	1.84	(1.40, 2.42)	1.55	(1.26, 1.90)	1.55	(1.26, 1.90)	1.36^b^	(1.11, 1.67)	1.38^b^	(1.12, 1.70)
Dissatisfied	Good	384	49.7	1.00	(referent)	1.00	(referent)	1.00	(referent)	1.00	(referent)	1.00	(referent)	1.00	(referent)	1.00	(referent)	1.00	(referent)
	Poor	293	64.8	1.92	(1.32, 2.80)	1.91	(1.32, 2.78)	1.34	(0.92, 1.97)	1.33	(0.92, 1.93)	1.23	(0.95, 1.59)	1.24	(0.96, 1.60)	0.98	(0.76, 1.26)	0.99	(0.76, 1.29)

Model 1 was adjusted for baseline age, sex, ethnicity, marital status, and number of people living in household.

Model 2: model 1 additionally controlled for depressive symptoms.

Model 3: model 1 additionally controlled for ADL and IADL.

Model 4: model 2 additionally controlled for ADL and IADL.

*n = 3275 for the 6–15-year model.

^a^Significant difference between “satisfied” and “dissatisfied” based on the χ^2^ test.

^b^Significant difference between “average” and “dissatisfied” based on the χ^2^ test.

Regarding financial satisfaction (Table 3), although none of the main effects was statistically significant in the 6–15-year full model, a higher predictive power of SRH for mortality was observed among those with the highest level of financial satisfaction, after controlling for ADL and IADL (model 3, HR = 1.44, 95% CI = 1.14, 1.82). This was significantly higher than the HR of 0.98 (95% CI = 0.76, 1.26) in the dissatisfied group. Similarly, the HR observed in the average group in model 3 was 1.36 (1.11, 1.67), which was also significantly higher than the HR for the dissatisfied group. A similar pattern was observed in model 4, which additionally adjusted for depressive symptoms. Again, no statistical difference was observed in HRs among the financial categories when the analysis was limited to a 5-year follow-up.

## DISCUSSION

SRH has been used as a health measure in research due to its predictive power for subsequent mortality. However, this generally high predictive power for subsequent mortality varies by SES group in younger age groups.^1^^–^^3^ The role of SES in health seems to be less apparent during old age, and it is therefore important to test whether the predictive power of SRH for subsequent mortality also varies by SES among elderly adults. We chose the 2 SES measures that are most widely used for elderly adults—educational attainment and subjective financial satisfaction—and found that the difference in predictive power across SES groups was still observable during old age. However, this greater predictive effect for the higher SES group was not observed when the analysis was limited to a 5-year follow-up.

Our results showed that deviations of SRH from true health status across different SES groups should not be overlooked in elderly respondents. SRH may be less likely to correspond to true health among elderly adults in lower SES groups as compared with those in higher SES groups. However, our results also suggest that such SES disparities may be of less concern when the follow-up period is short, as there was no significant difference in HRs across the SES groups in the present study when follow-up was limited to 5 years. In other words, the use of SRH as a predictor of 5-year mortality in elderly adults may yield similar results across SES groups. However, such similarity in the predictive power across SES groups disappears if SRH is used as a predictor of longer-term mortality.

To our knowledge, Regidor et al^8^ are the only group that specifically sampled elderly adults (age 60 years or older between 2000 and 2001). They, too, found a higher relative mortality risk among those with a higher education level. Our results differ from theirs in that we added a subjective SES measure that is often used to assess SES in elderly adults. It should be noted that the mechanism by which subjective SES (such as financial satisfaction) affects the predictive power of SRH on mortality may be different from that for education level. For example, education may be associated with knowledge, while financial satisfaction may be more associated with social position. Identification of these mechanisms is beyond the scope of this study.

In comparing our results with research using younger cohorts, our findings are consistent with those of Dowd and Zajacova,^1^ who also found that those in the highest education group had the highest HR for death. Our results, however, differ from those of Singh-Manoux et al,^3^ who observed that the predictive power of SRH for mortality weakens with increasing SES. They argued that this decrease in predictive power could be due to the composition of the age groups; their sample consisted of middle-aged adults.

There are special characteristics of the older age group that should be noted. First, it is reasonable to assume that elderly adults generally have more-apparent health problems. This explains why controlling for ADL and IADL lowered the hazard ratios. Second, the role played by socioeconomic status is more ambiguous during old age than when individuals are younger. Previous research has shown that the effect of education on health diminishes with increasing age and that this remains true even after controlling for selective survival effects.^25^ Nevertheless, our results show that SES still modifies the relationship between SRH and mortality during a longer follow-up period. This is consistent with the results of the study by Singh-Manoux et al,^3^ who, in a sample of middle-aged workers, separated their analysis into all-deaths and deaths during the first 10 years of follow-up. They also observed higher HRs during a shorter follow-up period, but the difference among participants with different education levels was weaker. One possible explanation for this is that, as compared with those with a lower SES, individuals with a higher SES rate their health status based on factors other than being closer to death. Research has indicated that SRH is directly contingent on social experience. It is likely that such experiences differ among SES groups.^26^ Thus, when there are obvious symptoms of poor health (which suggest that death is near), they may be equally recognized and incorporated in SRH by all SES groups. However, when the symptoms are subtle and may not immediately lead to death, higher SES groups may be better able to observe them.

One merit of this study is that we have data on ADL and IADL and hence can observe whether the interactive effect between SRH and the SES measures is channeled by these more objective health measures. Second, education level tends to be consistent during different periods of follow-up. This makes education (as opposed to occupation and income) a more appropriate measure of SES in the sample. Finally, we introduced a subjective measure of SES, which we argue better represents the SES status of elderly adults.

The limitations of this research should also be noted. First, this study did not take into account the possible cohort effect. Education may only partially reflect the SES of the sample because the proportion of participants with a high education level was low, and this may vary from cohort to cohort. Second, since the nationally representative survey used for this study consisted only of elderly adults from Taiwan, the findings cannot be generalized to other countries with different cultures. Studies have shown that factors other than a person’s actual physical condition, such as expectation and comparison, may also determine SRH and may be influenced by culture.^27^

In conclusion, our results are important because SRH has been widely used as a health measure in research on SES disparity in elderly adults. The validity of these studies relied on the validity of SRH as a measure of true health. One method of validation is to estimate the extent to which SRH predicts mortality. We found that regardless of whether education or subjective SES was used, the predictive power of SRH for mortality during old age varied across SES groups. However, this phenomenon was less of a problem when the follow-up period was shortened. Thus, with respect to future research, SRH is a better predictor of 5-year mortality than of longer term mortality.
